# System-agnostic prediction of pharmaceutical excipient miscibility via computing-as-a-service and experimental validation

**DOI:** 10.1038/s41598-024-65978-2

**Published:** 2024-07-02

**Authors:** Georgios S. E. Antipas, Regina Reul, Kristin Voges, Samuel O. Kyeremateng, Nikolaos A. Ntallis, Konstantinos T. Karalis, Lukasz Miroslaw

**Affiliations:** 1 Molecular Modelling Laboratory, Bahnhofplatz, 6300 Zug, Switzerland; 2 AbbVie Deutschland GmbH & Co. KG, Development Sciences, 67061 Ludwigshafen, Germany; 3 Azure High Performance Computing and Artificial Intelligence, Microsoft Switzerland, The Circle 02, 8058 Zurich, Switzerland

**Keywords:** Active pharmaceutical ingredient, Excipient, Surfactant, Placebo, Amorphous solid dispersion, Physical stability, Amorphous phase separation, Recrystallization, Solubility limit, Molecular dynamics, Molar free energy, Chemical potential, Computing as a service, Atomistic models, Computational methods, Pharmaceutics

## Abstract

We applied computing-as-a-service to the unattended system-agnostic miscibility prediction of the pharmaceutical surfactants, Vitamin E TPGS and Tween 80, with Copovidone VA64 polymer at temperature relevant for the pharmaceutical hot melt extrusion process. The computations were performed in lieu of running exhaustive hot melt extrusion experiments to identify surfactant-polymer miscibility limits. The computing scheme involved a massively parallelized architecture for molecular dynamics and free energy perturbation from which binodal, spinodal, and mechanical mixture critical points were detected on molar Gibbs free energy profiles at 180 °C. We established tight agreement between the computed stability (miscibility) limits of 9.0 and 10.0 wt% vs. the experimental 7 and 9 wt% for the Vitamin E TPGS and Tween 80 systems, respectively, and identified different destabilizing mechanisms applicable to each system. This paradigm supports that computational stability prediction may serve as a physically meaningful, resource-efficient, and operationally sensible digital twin to experimental screening tests of pharmaceutical systems. This approach is also relevant to amorphous solid dispersion drug delivery systems, as it can identify critical stability points of active pharmaceutical ingredient/excipient mixtures.

## Introduction

The majority of novel active pharmaceutical ingredients (API) fall into the low bioavailability BCS II or IV classes, characterized by poor water solubility and membrane permeability^1–4^. Bioavailability enhancement of these drugs may be addressed via amorphous API embedment in an excipient matrix toward formation of an amorphous solid dispersion (ASD)^4–7^, typically manufactured via hot melt extrusion (HME) or spray drying^8^. The ASD matrix normally consists of a hydrophilic polymer/surfactant (PS) mixture, with surfactants incorporated to promote API wettability and dissolution^9^.

However, although ASD offer some degree of API kinetic-stabilization^10^, their physical stability may be compromised by both (a) activated API nucleation and (b) activationless^11,12^ amorphous phase separation^13,14^, the latter leading to formation of API-rich clusters which promote API nucleation^15,16^. Both (a) and (b) eventually result in unwanted API recrystallization (AR)^6,17^. AR is thermodynamically favored if the intrinsic API solubility limit (SL) is exceeded^18^ and is accelerated by elevated storage temperatures and humidity uptake, causing matrix plasticization^14^ and further SL depression^16^. In fact, accelerated AR in the vicinity of the ASD binodal can be caused by sub-optimal PS selection at the pre-formulation stage, due to amorphous phase separation or system immiscibility; PS stability can be almost exclusively compromised by phase separation during HME^19^.

Typically, PS miscibility and stability are determined by pre-formulation differential scanning calorimetry (DSC) heating cycles^13,20,21^. Although definitive, such pre-formulation stress tests are also prohibitively time-consuming for efficient scans of the PS combinatorial space to be complete. Especially for solvent-free ASD formulation techniques such as HME, not only is it crucial to screen for the right PS composition ensuring miscibility/stability in the ASD but it is equally important to ensure that, at the high temperatures relevant to the process, the PS is miscible in the melt. This calls for manufacturing of several PS placebo batches of varying compositions, followed by lab characterization to identify the optimal PS composition. Such pre-formulation screening activities require significant amounts of PS material, resources for execution, and may take several days before the optimal PS composition can be established for further formulation development.

We reason that a viable strategy is pre-formulation miscibility/stability screening from computational first principles. Contrary to heuristic quantitative structure–activity schemes^10,20^, ab initio screening can system-agnostically yield critical stability (e.g., spinodal) points; the only input to the method are the component monomer and repeat unit structures, which are invariably known. Accordingly, here we discuss a massively parallel computing-as-a-service (CAAS) implementation, developed to be used as an unattended, solid solution pre-formulation digital twin. To exemplify the method, we present results for two PS systems, namely Vitamin E TPGS (surfactant)/Copovidone (polymer) and Tween 80 (surfactant)/Copovidone, the particular polymer having been chosen due to its frequent use in ASD formulations. The method is based on the calculation of Gibbs free energy profiles via molecule annihilation^22^ and, aside from PS, the method is readily applicable to any solid solution including dry and hydrated ASD.

## Results

We first present results from the molecular modelling method and then, proceed to discuss the experimental validation of these predictions.

### Molecular modelling

#### CAAS setup

To enable on demand simulations, we deployed a high-performance computing (HPC) cluster in Microsoft (MS) Azure. The Azure Cyclecloud (CC) service was used to control the HPC cluster, composed of a head node and a number of compute nodes. For security and compliance reasons, multiple user access to the target system was provided via a bastion service, a jump server and multi-factor authentication. The entire infrastructure was created automatically by custom Azure Resource Manager (ARM) templates. A high-level diagram of this architecture is shown in Supplementary Fig. 1.

#### Construction and parametrization of model components

The Vitamin E TPGS/ Copovidone and Tween 80/Copovidone placebo systems considered for simulation are designated as VIE and T80, respectively (Table 1). For each of the Copovidone polymer repeat unit, and Vitamin E TPGS and Tween 80 surfactant monomers, we computed CHARMM-compatible force field parameters via D4 dispersion-corrected^23^ density functional theory (DFT) at the PBE0 level.

**Table 1:** Model components. Molecular weights shown are based on model structures.

Name	Designation	Molecular weight (g/mol)	Skeletal structure
D-α-Tocopherol polyethylene glycol succinate	Vitamin E TPGS	1984.513	
Polyoxyethylene (80) sorbitan monooleate (Polysorbate 80)	Tween 80	1309.654	
Polyvinylpyrrolidone-co-vinylacetate (Kollidon VA64)	Copovidone	3818.088	

#### Molecular dynamics and free energy perturbation

**Figure 1:**
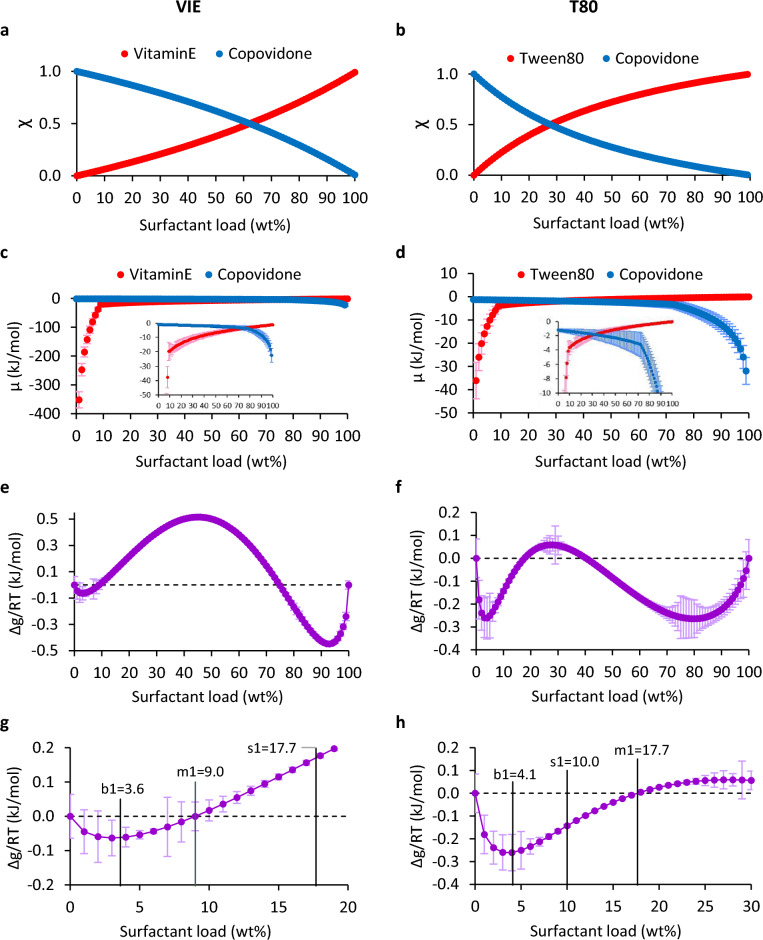
Calculated thermodynamic descriptors vs. surfactant load at 180 °C. For the VIE and T80 systems respectively: (**a**,**b**) surfactant and polymer molar fraction, χ. (**c**,**d**) surfactant and polymer chemical potential, μ. Insets focus on the region of surfactant-polymer μ intercept. (**e,f**) molar Gibbs free energy, Δg. (**g,h**) critical Δg points in the polymer-rich region: b1 binodal, s1 spinodal and m1 mechanical/chemical mixture limit. For the VIE system, the critical points in the surfactant-rich region (not shown for clarity) were m2 = 74.8, s2 = 79.0 and b2 = 93.3, while for the T80 system, these points (also not shown) were m2 = 40.3, s2 = 50.8 and b2 = 80.2.

To determine placebo miscibility under the effect of thermal motion, we constructed amorphous supercells of 10^5^ atoms in average size with surfactant loads ranging from 0 to 100 wt% at steps of 1 wt% according to VIE and T80 model mol fraction vs. wt% content (Fig. 1a,b, respectively), the latter estimated from simple mixing of component molecular weights (Table 1). Each supercell was then subjected to isothermal-isobaric (NPT) molecular dynamics simulations (MD) under periodic boundary conditions, gradually converging system density at a temperature of 180 °C (which is relevant to ΗΜΕ process) and calculating component chemical potentials, μ, by worker-parallelized monomer annihilation via the single-topology free energy perturbation (FEP)22 method. Computing resource allocation per simulation phase, production MD execution, MD time per FEP λ-window, MD/FEP convergence monitoring, post-production trajectory analysis, and extraction of molecular descriptors run fully unattended. Individual MD/FEP simulations scaled well across 100 to 200 CPU 2.25 GHz cores, depending on supercell size and chemical environment, achieving an aggregate CPU utilization which exceeded 90%. Final CPU allocation was approx. 15,000 cores per system. FEP worker parallelization combined with high CPU utilization resulted in simulation completion of approx. 5 wall-clock days and a consumption of approx. 630 VM-days per system. All simulations used Microsoft Azure spot VM instances.

#### Reconstruction of molar Gibbs free energy profiles

During FEP sampling, the compound chemical potential was periodically sampled and FEP simulations were considered complete when the chemical potential standard deviation, σ_μ_, fit to a polynomial spline exceeding an R^2^ value of 0.99; FEP final spline fit for both systems is shown in Supplementary Fig. 2. The resulting chemical potentials of the VIE and T80 models are shown in Fig. 1c,d, from which we computed the molar Gibbs free energy of mixing, Δg, as

1 $$\Delta {\text{g}}/{\text{RT}} = \Delta {\text{G}}/{\text{nRT}} =\upchi \left( {\upmu _{{\text{s}}} -\upmu _{{\text{s}}}^{0} } \right) + \left( {1 -\upchi } \right)\left( {\upmu _{{\text{p}}} -\upmu _{{\text{p}}}^{0} } \right)$$

where ΔG is the free energy of mixing, R is the gas constant, T is the FEP temperature, n is the number of moles in the system, χ is the mol fraction of the surfactant, subscripts s and p refer to surfactant and polymer respectively, and superscript 0 refers to the chemical potential reference state which is taken to be the pure phase. Calculated Δg profiles based on Eq. (1) are shown in Fig. 1e,f, for the VIE and T80 models, respectively.

Three types of critical points were, then, distinguished: (i) binodal (single phase) points, at Δg’ = 0 and Δg’’ > 0, (ii) spinodal (inflection) points at Δg’’ = 0 and (iii) mechanical/chemical mixture limits, at Δg = 0, where single and double prime symbols respectively indicate first and second derivatives with respect to surfactant molar fraction. By numerical differentiation of the Δg datasets we located the lower binodal points for the VIE and T80 at 3.6 wt% (s1 in Fig. 1g) and 4.1 wt% surfactant (s1 in Fig. 1h), respectively. However, the limit of physical stability of the VIE system was determined to be a mechanical/chemical mixture threshold at 9.0 wt% surfactant (m1 in Fig. 1g). Similarly, the limit of physical stability of the T80 system was the lower spinodal point (s1 in Fig. 1h), leading to phase separation above 10.0 wt%, prior to the system transitioning into a mechanical mixture above 17.7 wt% surfactant (m1 in Fig. 1h). It is important to determine the extent by which FEP-derived stability limits are reproductive of results from an analytical activity model, as the latter constitutes orthogonal computation to MD/FEP and, by design, satisfies the Gibbs–Duhem equation for symmetric solutions. The condition for thermodynamic instability is

2 $$\Delta {\text{g}}^{\prime \prime } = \frac{{{\text{d}}^{2} \left( {\Delta {\text{g}}^{{\text{I}}} + \Delta {\text{g}}^{{\text{E}}} } \right)}}{{{\text{d}}\upchi ^{2} }} < 0$$

where $\Delta {\text{g}}^{{\text{I}}}$ and $\Delta {\text{g}}^{{\text{E}}}$ are the ideal and excess parts of Δg, defined as

3 $$\Delta {\text{g}}^{{\text{I}}} = {\text{RT}}\left[ {\upchi \ln \left(\upchi \right) + \left( {1 -\upchi } \right)\ln \left( {1 -\upchi } \right)} \right]$$

4 $$\Delta {\text{g}}^{{\text{E}}} = {\text{RT}}\left[ {\upchi \ln \left( {\upgamma _{{\text{s}}} } \right) + \left( {1 -\upchi } \right)\ln \left( {\upgamma _{{\text{p}}} } \right)} \right]$$

and γ_s_ and γ_p_ are the surfactant and polymer activity coefficients, respectively. The ideal and excess Δg contributions for both systems are shown in Supplementary Fig. 3 while the Δg difference profile is shown in Supplementary Fig. 4. Dividing Eqs. (3) and (4) with RT and substituting into Eq. (2), we obtain the spinodal points as satisfying

5 $$\frac{1}{{\upchi \left( {1 -\upchi } \right)}} + \frac{{{\text{d}}^{2} \left[ {\upchi \ln \left( {\upgamma _{{\text{s}}} } \right) + \left( {1 -\upchi } \right)\ln \left( {\upgamma _{{\text{p}}} } \right)} \right]}}{{{\text{d}}\upchi ^{2} }} = 0$$

To evaluate Eq. (5), we first calculated activity coefficients, γ, from component μ as

6 $$\upgamma = \frac{{e^{{\frac{{\upmu -\upmu ^{0} }}{{{\text{RT}}}}}} }}{\upchi }$$

**Figure 2:**
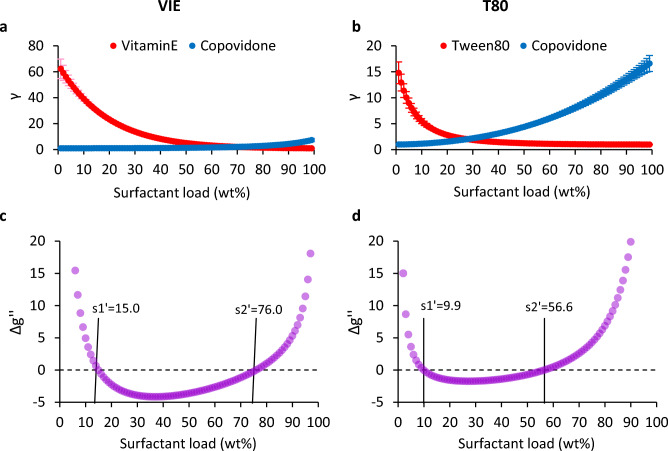
Margules one-parameter model stability limits from FEP γ at 180 °C. For the VIE and T80 systems respectively: (**a**,**b**) surfactant and polymer FEP-derived activity coefficients (γ). (**c,d**) surfactant and polymer $\Delta {\text{g}}^{\prime \prime }$ curves and spinodal points (standard deviation bars are not shown for clarity). Agreement with FEP-derived limits (calculated as, 1-│s1’-s1│/s1) was: for VIE (Figs. 1g vs. 2c) s1’ vs. s1 0.85, s2’ vs. s2 0.96, for T80 s1’ vs. s1 0.99, s2’ vs. s2 0.89.

The resulting γ values for the VIE and T80 systems are shown in Fig. 2a,b, respectively. Next, we fitted the γ datasets to Margules one-parameter surfactant, A_s_, and polymer, A_p_, constants expressed as^24^

7 $${\text{A}}_{{\text{s}}} = \frac{{\ln \left( {\upgamma _{{\text{s}}} } \right)}}{{\left( {1 -\upchi } \right)^{2} }}$$

8 $${\text{A}}_{{\text{p}}} = \frac{{\ln \left( {\upgamma _{{\text{p}}} } \right)}}{{\upchi ^{2} }}$$

acquiring A_s_ = 4.18, A_p_ = 2.09 for VIE and A_s_ = 2.84, A_p_ = 2.83 for T80. Solving Eqs. (7), (8) for γ_s_ and γ_p_ and substituting it into Eq. (5), we derived the $\Delta {\text{g}}^{\prime \prime }$ plots of Fig. 2c,d, for VIE and T80 respectively. Spinodal point agreement factors between the FEP- and Margules-predicted limits ranged between 0.85 and 0.99 (Fig. 2 caption).

### Solid dispersion formulations

To assess the accuracy of the computed stability limits, we experimentally determined the miscibility limits of the VIE and T80 samples, as follows.

#### Sample preparation by HME

**Figure 3:**
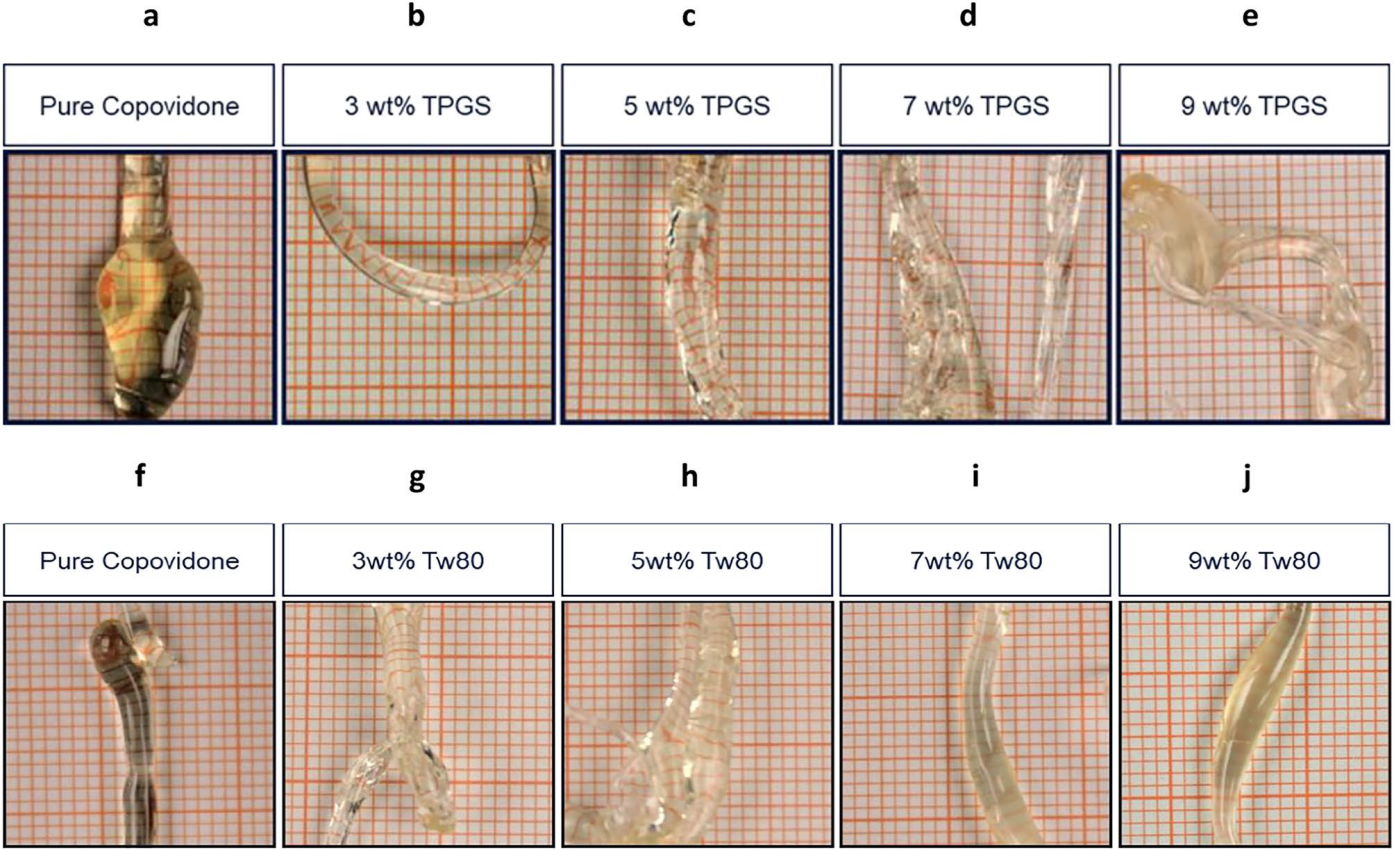
Visual appearance of extrudate samples. (**a**–**e**) Copovidone/Vitamin E TPGS and (**f**–**j**) Copovidone/Tween 80.

Binary PS systems were formulated by HME from Copovidone blends with Vitamin E TPGS and Tween 80 at 3, 5, 7 and 9 wt% surfactant levels. The extrudates were visually examined for transparency, as a means of preliminary investigation of component miscibility (Fig. 3). The Vitamin E TPGS extrudates only appeared turbid at 9% loading (Fig. 3e), while the Tween 80 samples appeared hazy at 7% and completely turbid at 9% loading (Fig. 3i,j, respectively), suggesting immiscibility between the components.

#### DSC

**Figure 4:**
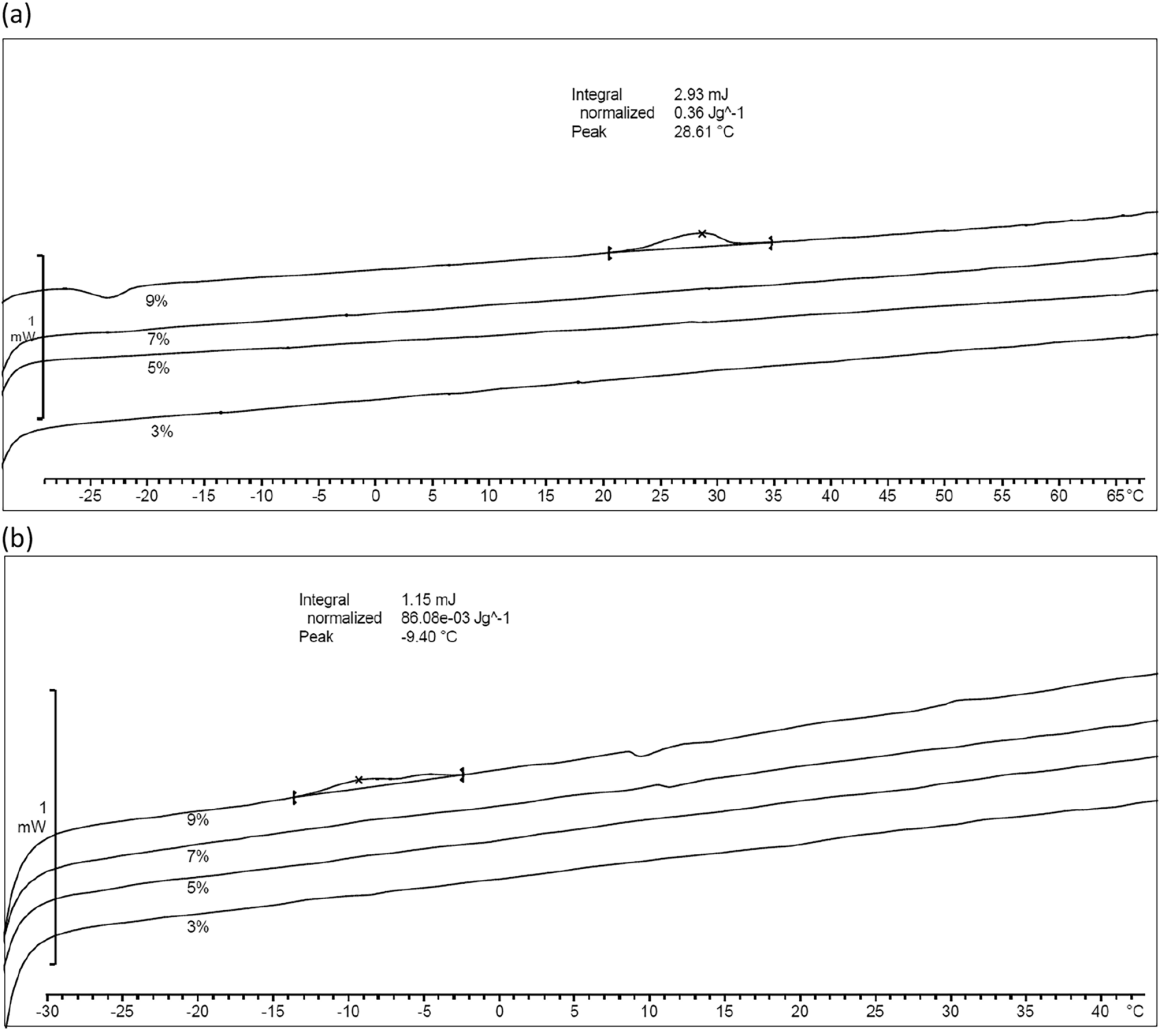
DSC thermograms after 30 min annealing at 180 °C and quenching. Samples based on (**a**) Copovidone/Vitamin E TPGS and (**b**) Copovidone/Tween 80 with 9, 7, 5 and 3 wt% surfactant loads, respectively.

To further refine miscibility limits, we milled the extrudate samples and examined them with DSC cycling (stress experiments) between -60 and 180 °C. DSC investigation results are listed in Table 2 indicating phase separation at Vitamin E TPGS loads of 7 and 9 wt% surfactant. For the Tween 80 system, the DSC results confirmed that phase separation occurred at 9 wt% surfactant load; we note that although the 7 wt% load sample appeared hazy at visual inspection, DSC cycling did not confirm phase separation. Figure 4 depicts the DSC thermograms after annealing of the milled samples at 180 °C and subsequent quenching; for both systems a second phase was detected as peak at 9 wt% surfactant loading, indicating phase separation. The Vitamin E TPGS system stability limit 7 wt% vs. 9 wt% is responsive to the tempering protocol, cycling vs. annealing, which may be attributable to the fact that its destabilization mechanism was computationally determined to be a mechanical/chemical mixture limit compared to Tween 80 which was determined to be a spinodal limit.

**Table 2:** Detection of phase separation after DSC thermal cycling.

Surfactant	Surfactant load (wt%)	Phase separation
Vitamin E TPGS	3	No
	5	No
	7	Yes
	9	Yes
Tween 80	3	No
	5	No
	7	No
	9	Yes

## Discussion

We established tight agreement between the computed stability limits of 9.0 and 10.0 wt% vs. the experimental 7 and 9 wt% based on DSC annealing at 180 °C, for the Vitamin E TPGS and Tween 80 systems, respectively, and identified different destabilizing mechanisms applicable to each system.

It is noteworthy that the VIE and T80 systems have markedly different surfactant-polymer (χ = 0.5) intercept positions (63 wt%, for VIE in Fig. 1a and 28 wt% for T80 in Fig. 1b) which are directly reflected to $\Delta {\text{g}}^{{\text{I}}}$ minima positions (62 wt%, for VIE in Supplementary Fig. 3a and 28 wt% for T80 in Supplementary Fig. 3b) through Eq. (3). Additionally, both the VIE $\Delta {\text{g}}^{{\text{E}}}$ and Δg maxima are located at 55 wt% surfactant (Supplementary Fig. 3a), while both the T80 $\Delta {\text{g}}^{{\text{E}}}$ and Δg maxima are located at 28 wt% surfactant (Supplementary Fig. 3b). Given that the T80 mixture is practically symmetric (A_s_ = 2.84, A_p_ = 2.83 from Eqs. (7) and (8), respectively), the results suggest that the load point of the χ intercept is correlated with the stationary point inside the miscibility gap. On the contrary, the position of the χ intercept for VIE (non-symmetric solution, A_s_ = 4.18, A_p_ = 2.09) does not coincide with the miscibility gap stationary point. An analogous argument can be made for μ (77 wt% for VIE in Fig. 1c inset and 33 wt% for T80 in Fig. 1d inset) and γ (70 wt%, for VIE in Fig. 2a and 28 wt% for T80 in Fig. 2b) intercept points. Therefore, no priori conclusion may be safely drawn with respect to stability limits solely based on the χ, μ or γ intercept points.

Another point of interest raised by the calculations is the ten-fold difference in surfactant μ values (Fig. 1c vs. d), despite comparable (starting structure) internal energies of 2200 vs. 1550 kJ/mol for Vitamin E TPGS and Tween 80, respectively; this observation suggests that the nature of the energy difference in Supplementary Fig. 4 is mainly owing to entropic contributions. As a result, we may assume that entropy differences determine the type of instability mechanism (transition to a mechanical mixture for VIE and transition into the spinodal for T80) rather than stability limit positions (these lie at almost identical load points for both systems).

As this approach is not only valid for binary mixtures of polymer and surfactant but would also be true for API polymer mixtures and their demixing and stability behavior, this holds promise as a tool for early phase formulation design.

## Conclusions

We portrayed that purpose-built, massive parallelization of ab initio FEP on CAAS was able to pinpoint Vitamin E TPGS/Copovidone (VIE) and Tween 80/Copovidone (T80) placebo miscibility (stability) limits to within an accuracy of 2 and 1 wt% surfactant from experimental VIE and T80 values, respectively. Furthermore, the method indicated the presence of different instability mechanisms for the two systems, i.e., transition to a mechanical mixture for VIE and transition to the spinodal region for T80. Fully based on first principles and with a modest computational cost footprint, this method may serve as a digital twin to pharmaceutical pre-formulation screening and high-throughput system selection for both PS and ASD.

## Materials and methods

### Molecular modelling

All calculations were run on the ActiveRank cloud service, implemented on MS Azure and available on demand.

#### CAAS setup

A virtual network was created in an active MS Azure subscription, hosting login and compute VM, and an MS Azure Bastion service was activated to allow secure VM user login. Access to whitelisted domains for data exchange was enabled via Network Security Group (NSG) rules. The MS Azure CC orchestration server and storage container were launched using a VM Managed Identity and separate CC level user account RSA keys were created for login. A custom operating system image with Molecular Modeling Laboratory software was transferred to CC via a SAS key, containing all business logic (simulation) software, followed by assignment of HBv2 compute VMs (each HBv2 VM featuring 120 AMD EPYC 7002 CPU cores, 4 GB RAM per core and 350 GB/s memory bandwidth, interconnected with 200 Gb/s Mellanox HDR Infiniband, enabling high scalability across multiple nodes). Excluding image transfer and backup policy settings, MS Azure infrastructure was automatically created via ARM MML templates.

#### Monomer parametrization

Monomer-specific potential parameters were calculated via D4 dispersion-corrected^23^ DFT relaxation at the PBE0 level, driven by a proprietary engine written in C++, performing potential energy surface scans and parallelized for execution on MS Azure. The resulting parameters were then used to derive CHARMM-compatible force field parameters for surfactant monomers and excipient repeat units. For each component structure, a DFT optimization was first performed to derive the ground state geometry. Next, structures were distorted both isotopically (all atom bonds expanded/contracted by the same amount) and anisotropically (all atoms translated in all principal directions by the same amount). Then, the computed electronic energy was mapped to CHARMM-compatible bonds/angles/dihedrals/impropers energies, via stepwise parameter refinement. Starting from a draft parameter set, consecutive iterations were performed until the difference between DFT and classical energy was less than 1 meV. The same procedure was followed for the dispersion energy, extracting ε and σ for the Lennard–Jones (dispersion) potential. For intermolecular dispersion interactions, a geometric mixing rule was used, as $\upvarepsilon _{{{\text{ij}}}} = \sqrt {\upvarepsilon _{{\text{i}}}\upvarepsilon _{{\text{j}}} }$ and $\upsigma _{{{\text{ij}}}} = \sqrt {\upsigma _{{\text{i}}}\upsigma _{{\text{j}}} }$. Finally, a force-matching method was applied at 100 and 300 °K, by which we subjected the systems to loops of DFT-MD for at least 1000 1 fs timesteps, followed by partial charge and dispersion parameter refinement, until a tolerance of 1.0e−3 meV/Å was reached between DFT and classical forces.

#### MD simulations

MD simulations were driven by a proprietary calling routine written in C++, parallelized for execution on MS Azure. All simulations were carried out in the NPT ensemble at 1.01325 bar, maintained by the isotropic Nosé-Hoover Langevin piston method^25^, under periodic boundary conditions. Temperature control was via Langevin^26^ coupling. Electrostatic interactions were computed via the particle mesh Ewald method^27^. Short-range interactions were truncated at SRO limits determined from pair distribution function data^28–41^. The equations of motion were integrated with the velocity Verlet algorithm at a time step of 1 fs.

#### FEP simulations

Chemical potentials were calculated by component monomer annihilation via the single-topology FEP^22^ method. In the ActiveRank implementation, Gibbs free energy differences between the two end states were computed as the sum of gradual decoupling of the electrostatic and LJ interactions across separate λ windows, the number and simulation time spent on each of which was set dynamically based on energy convergence vs. λ. Typical values were 20 λ windows with an MD time of 10 ns per window. Partial charges as well as the LJ potential σ and ε parameters were scaled linearly with λ. All van der Waals interactions included a soft-core potential^42^. Free energy differences between successive λ values were calculated based on Bennett’s acceptance ratio method^43^.

### Experiments

#### Sample preparation by HME

Copovidone (Kollidon VA64, BASF) was first granulated with each of Vitamin E TPGS (D-α-Tocopherol polyethylene glycol succinate, Gattefosse) and Tween 80 (Polysorbate 80, Merck KG, Darmstadt, Germany) surfactants using a Thermomix (Vorwerk), followed by sieving with 1 mm mesh. Mixtures with surfactant loadings of 3, 5, 7 and 9 wt% were then prepared. The sieved binary mixtures were extruded using a Rondol MicroLab 10 mm Twin Screw extruder (Rondol Technology Ltd, UK) at 200 rpm screw speed. Extrusion was performed at 180 °C with a feed rate of 80 to 120 g/h. The melt temperature at the die was measured by an infrared camera (Optris PI 200, Optris GmbH, Germany) and the recorded temperature was 182.5 ± 2.0 °C. The extrudate samples were milled 3 times for 10 s each using the IKA A10 Basic Mill (IKA-Werke) and subsequently passed through a Kressner sieve with a 250 µm mesh size.

#### DSC measurements

The milled extrudate samples were analyzed by DSC using DSC 1 or DSC 300 + (Mettler Toledo). The samples (5–12 mg) were weighed into 40 µl aluminum pans with pierced lids. DSC measurements were performed under nitrogen flow. DSC thermal cycling stress tests were performed as follows. Samples were cooled from 25 to − 60 °C at a rate of 10 °C/min, heated from − 60 to 180 °C, and cooled back to − 60 °C at a rate of 1.5 °C/min. The heating and second cooling ramps were repeated before finally heating to 180 °C at a rate of 10 °C/min. DSC annealing studies were performed as follows: samples were heated to 180 °C at a rate of 100 °C/min, annealed for 30 min at 180 °C, followed by rapid cooling to − 40 °C at a rate of 100 °C/min and reheated to 180 °C at a rate of 10 °C/min.

### Supplementary Information

Supplementary Information.

## Data Availability

The datasets used and/or analyzed during the current study are available from the corresponding author on reasonable request.
