# Supplementary material for: System-agnostic prediction of pharmaceutical excipient miscibility via computing-as-a-service and experimental validation
Source: Sci Rep. 2024 Jul 2;14:15106. doi: 10.1038/s41598-024-65978-2 (PMC11219749; doi:10.1038/s41598-024-65978-2)
Supplement: Supplementary file 1 — Supplementary Information. [file 41598_2024_65978_MOESM1_ESM.docx]

**Supplementary information**


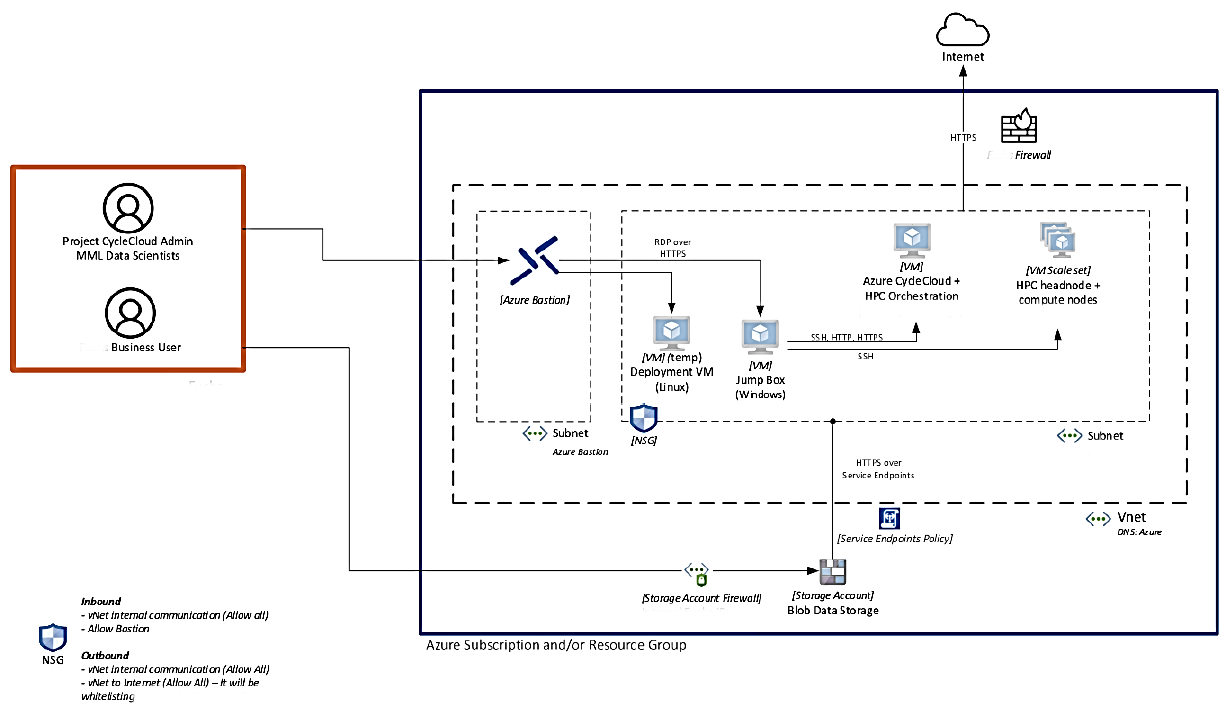


**Supplementary Figure 1**. **High-level diagram of MS Azure infrastructure components as implemented in ActiveRank**. The setup comprises an isolated sandbox with restricted user access based on NSG rules. Users connect to jump hosts via the Bastion service without directly exposing the head and compute nodes to public internet. Both the jump hosts and head node have whitelisted outbound connections for data transfer. The sandbox is connected to a storage account for backup.

| **a** | **b** |
| --- | --- |

**Supplementary Figure 2. Chemical potential standard deviation,** **σ_μ_ vs. surfactant load.** Open circles are σ_μ_ and solid lines are the lowest-order polynomial splines which fit σ_μ_ data with an R^2^ higher than or equal to 0.99. (a) VIE system. Vitamin E TPGS spline y = 2E-06*w^6^ - 0.0007*w^5^ + 0.096*w^4^ - 6.5007*w^3^ + 229.3*w^2^ - 3983.7*w + 29062, R² = 0.9900. Copovidone spline y = 2E-07*w^6^ - 5E-05*w^5^ + 0.0045*w^4^ - 0.2055*w^3^ + 4.416*w^2^ - 25.972*w + 338.37, R² = 0.9964. (b) T80 system. Tween 80 spline y = 6E-07*w^6^ - 0.0002*w^5^ + 0.026*w^4^ - 1.7752*w^3^ + 63.236*w^2^ – 1113*w + 8096.9, R² = 0.9906. Copovidone spline y = 2E-07*w^6^ - 6E-05*w^5^ + 0.0055*w^4^ - 0.2509*w^3^ + 5.3913*w^2^ - 31.708*w + 266.17, R² = 0.9964, where w is surfactant load (wt.%).

| **a** | **b** |
| --- | --- |

**Supplementary Figure 3. Ideal and excess parts of the molar Gibbs free energy vs. surfactant load.** (a) VIE system. The ${\Delta g}^{I}$minimum and ${\Delta g}^{E}$maximum are located at 62 and 55 wt.% surfactant, respectively. (b) T80 system. The ${\Delta g}^{I}$minimum and ${\Delta g}^{E}$maximum are both located at 28 wt.% surfactant.

**Supplementary Figure 4. T80-VIE molar Gibbs free energy difference**.
